# Predicting the Reliability of Drug-target Interaction Predictions with Maximum Coverage of Target Space

**DOI:** 10.1038/s41598-017-04264-w

**Published:** 2017-06-19

**Authors:** Antonio Peón, Stefan Naulaerts, Pedro J. Ballester

**Affiliations:** 1 Centre de Recherche en Cancérologie de Marseille (CRCM), Inserm, U1068, Marseille, F-13009 France; 2 CNRS, UMR7258, Marseille, F-13009 France; 3 Institut Paoli-Calmettes, Marseille, F-13009 France; 4 Aix-Marseille University, UM 105, F-13284 Marseille, France

## Abstract

Many computational methods to predict the macromolecular targets of small organic molecules have been presented to date. Despite progress, target prediction methods still have important limitations. For example, the most accurate methods implicitly restrict their predictions to a relatively small number of targets, are not systematically validated on drugs (whose targets are harder to predict than those of non-drug molecules) and often lack a reliability score associated with each predicted target. Here we present a systematic validation of ligand-centric target prediction methods on a set of clinical drugs. These methods exploit a knowledge-base covering 887,435 known ligand-target associations between 504,755 molecules and 4,167 targets. Based on this dataset, we provide a new estimate of the polypharmacology of drugs, which on average have 11.5 targets below IC_50_ 10 µM. The average performance achieved across clinical drugs is remarkable (0.348 precision and 0.423 recall, with large drug-dependent variability), especially given the unusually large coverage of the target space. Furthermore, we show how a sparse ligand-target bioactivity matrix to retrospectively validate target prediction methods could underestimate prospective performance. Lastly, we present and validate a first-in-kind score capable of accurately predicting the reliability of target predictions.

## Introduction

Target deconvolution of phenotypic screening hits^1^ consists of identifying the macromolecular targets of small molecules exhibiting some kind of phenotypic activity (e.g. whole-cell activity)^2^. It is a prerequisite to gain mechanistic understanding of observable activity and has proven helpful for drug development^3, 4^. Indeed, the combination of phenotypic screening with target deconvolution constitutes an attractive alternative strategy for the discovery of molecularly targeted therapies. However, reliable computational methods for target prediction, also known as target fishing or polypharmacology prediction^5–9^, are crucial for this application. Target prediction tools are also used to predict drug side-effects^10^ and drug repositioning opportunities^9^.

The need for target prediction methods is exacerbated by the resurgence of phenotypic drug discovery^11–13^, as this trend has boosted the availability of new hits whose phenotypic activities are still to be explained mechanistically. A landmark study^3^ has shown that, despite a more intense focus on target-based drug discovery, most first-in-class drug approvals come from phenotypic screens. This realisation has contributed to many more research projects using this type of screens (e.g. large-scale empirical screening projects on cancer cell lines^14–18^ or pathogen cultures)^19, 20^. In turn, more phenotypic data has resulted in more accurate *in silico* models to predict new hits, whether this prediction is done from the chemical structure of molecules^21–24^ or more recently complemented with molecular profiles characterising the system in which the phenotype was measured^25–27^. Moreover, webservers for prospective virtual screening, which implement methods able to identify purchasable molecules with the same phenotypes as their template^28^, are now freely-available^29, 30^.

Computational methods for target prediction can be classified into two broad categories^7^: target-centric and ligand-centric. Target-centric methods build a predictive model for each target, which is used to estimate whether the molecule of interest has activity against the target. Afterwards, this query molecule is evaluated by each of these models to provide its set of predicted targets. Each method adopts a particular model type: supervised learning (e.g. Naïve Bayes Classifier^20, 31^, TAMOSIC^32^, Kernel Classifiers)^33^, unsupervised learning (e.g. SEA^34^, SuperPred^35^, ChemProt-2.0)^36^ or structure-based (e.g. TarFisDock^37^, INVDOCK^38^, PharmMapper)^39^. On the other hand, ligand-centric methods are based on calculating the similarity of a very large number of target-annotated molecules to the query molecule. This nomenclature is different from that employed by target-centric methods, where query and database molecules are generally referred to as test and training sets, respectively. There are fewer methods in the ligand-centric category and these are based on molecular similarity^40^ (e.g., ChemMapper^41^, ElectroShape Polypharmacology server)^42^ or on the similarity of bioactivity spectra (e.g. COMPARE)^18^. It is worth noting that not all methods employing molecular similarity are ligand-centric. This is the case of TAMOSIC^32^, which learns the optimal similarity cut-off for each target with at least 30 cognate ligands, and SEA^34^, which only builds a statistical model for a target if it is characterised by at least five samples (ligands).

As discussed in a previous study^7^, we are interested in ligand-centric target prediction methods because they provide the maximum coverage of the target space for a given data set. This is an advantage over target-centric methods, which can only evaluate the much smaller set of targets for which a predictive model can be built. There is an implicit trade-off here: one can make target-centric methods more predictive by only considering targets with a higher number of cognate ligands at the cost of reducing the number of targets that the method can possibly predict. Another advantage of ligand-centric methods is that they naturally lend themselves to investigate how performance depends on the considered query^7^. In that study^7^, we explained that prior validations for ligand-centric methods have resorted to using benchmarks borrowed from virtual screening, rather than actually building a benchmark suitable to measure performance at target prediction. To fulfil this unmet need, we constructed such a benchmark and thus could establish how the performance of ligand-centric methods depends on various factors. For example, predicting the targets of clinical drugs is far more challenging than predicting the targets of non-drug molecules^7^. Here we aim instead at improving the performance of ligand-centric methods for target prediction using this recently-built benchmark. With this purpose, we will search for the most suitable molecular similarity technique among those freely-available and strongly increase the amount of data exploited by the method. Most importantly, we propose and validate a first-in-kind method to predict how reliable ligand-centric target predictions are. The latter is highly beneficial in practice, as focusing experimental confirmatory tests on the most reliable predictions will lead to much higher hit rates.

## Results and Discussion

### Experimental design

**Figure 1:**
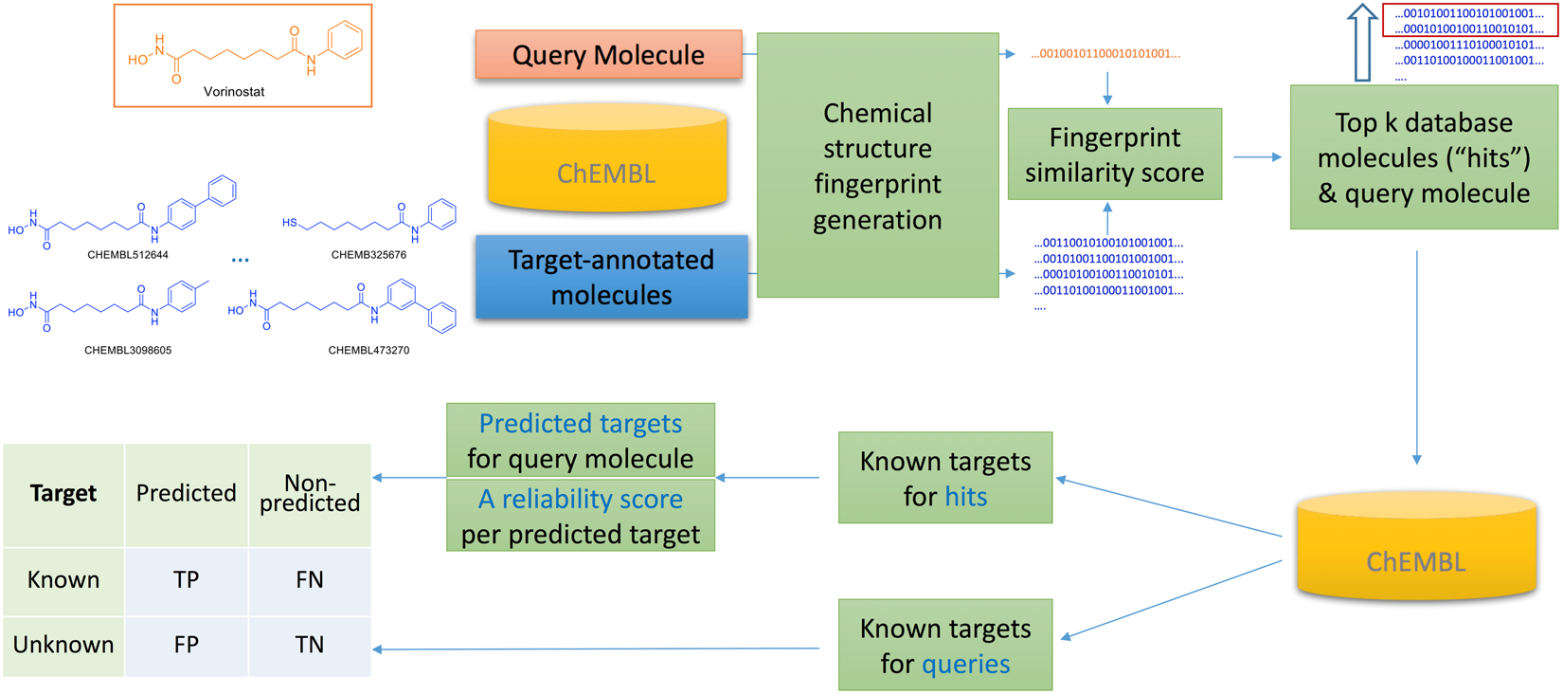
Ligand-centric target prediction workflow. The selected molecular similarity method is used to find the top k most similar database molecules to the query molecule (vorinostat in this illustrative example). Known targets for the query and the top k hit molecules are retrieved from the ChEMBL database. A novel method is introduced to assign a reliability score for each query-target association prediction based on the proportion of the query’s top hits binding to the predicted target. Lastly, the known targets of the query molecule permit measuring the predictive performance of the method at each reliability level in this binary classification problem.

Figure 1 illustrates how the molecular targets of a query molecule are predicted with a ligand-centric target prediction method and how the performance of such a prediction is measured. In a nutshell, once the chemical structure fingerprint and associated similarity score is selected (see the Methods section), the similarity scores between the query molecule and each of the database molecules are calculated. Similarity scores are employed to identify the top k molecules closest to the query molecule (an approved drug out of the 745 considered in this study). Next, target annotations are extracted from the ChEMBL database^43^ in order to determine the known targets for the query as well as those for the top k hits. The method uses the known targets for the top k hit molecules to predict the targets for the query molecule as well as estimate the reliability of each predicted target. Lastly, the known targets of the query molecule permit measuring the predictive performance of the target prediction method in this binary classification problem.

As usual, predicted targets for the query molecule will be divided into the four categories of the confusion matrix: TP is the number of true positive predictions (the predicted target is a known target of the query molecule), TN is the number of true negatives (the target was not predicted and is not known to be a target), FP is the number of false positives (the predicted target is not known to be a target, i.e. a false discovery or Type I error) and FN is the number of false negatives (the target was not predicted and it is actually a target, i.e., missed discovery or Type II error). From these quantities, we will calculate five performance metrics per query molecule. Accuracy (ACC) is the proportion of correct predictions. Precision (PR) is the proportion of correctly predicted targets, i.e. how many of the predicted targets are known to be true targets of the query molecule. Recall (RC) accounts for the proportion of known true targets that the method has missed. The Matthews Correlation Coefficient (MCC) captures both types of error in a single metric, with higher values being better up to +1 (perfect classification). Lastly, the Number of Predicted Targets (NPT) will be also reported to investigate how this quantity varies with the method’s control parameter k. Average values across the performed queries will be labelled with the prefix “Av” in front of the metric (e.g. AvMCC). The Methods section further specifies how each of these metrics is calculated from TP, TN, FP and FN.

Lastly, it is important to bear in mind that a false positive occurs when the predicted target is not known to be a target of the query molecule, which is not at all the same as not being a target of that molecule. This unavoidable uncertainty is due to ChEMBL bioactivity matrices being sparsely populated and its impact will be discussed later.

### Expanding ligand-target knowledge-base

We have previously performed a critical assessment of the current performance of ligand-centric methods for target prediction based on a ligand-target knowledge-base^7^. We found out that the targets of approved drugs are generally much harder to predict than those for other types of molecules. As virtually all target prediction methods employ test sets that are not entirely formed by approved drugs, their performance on this important type of query molecules may be overestimated.

It is both possible and beneficial to expand this knowledge-base by relaxing the data inclusion criteria. For instance, Mugumbate and co-workers^44^ retrieved targets that were proteins and had a target confidence scores of at least 7. This is a score in ChEMBL that shows the level of confidence in the target assignment to the ligand (scores 7, 8 and 9 indicate direct assignment to protein complexes, homologous single protein and single protein, respectively). In addition, there are many ligand-target associations supported by threshold-like experimental values such as IC_50_ < 1 µM, which were missed in Peon *et al*.^7^ by only using the relation ‘=‘ in the query, but are logically better than 10 µM. Furthermore, we have observed that the functional assay type also contains bioactivity data for molecular targets. Lastly, data from PubChem confirmatory assays were not included either (bioactivity type ‘Potency’). Briefly, in this study ligand-target associations are those ligand-target pairs passing the following filters: (i) the activity value was better than 10 µM using published relation “=” or “<”, (ii) the assay type was binding and functional, (iii) the bioactivity type was IC_50_, K_i_, EC_50_, K_d_ or Potency and (iv) the target confidence score was at least 7.

These data inclusion criteria lead to 887,435 known ligand-target associations in the knowledge-base, with 504,755 database molecules to screen annotated with 4,167 targets verifying the above requirements. This represents a substantial enhancement with respect to the knowledge-base used in our previous study^7^: 216% more ligand-target associations (from 281,270 to 887,435), 40% more targets (from 2,982 to 4,167) and 175% more database molecules (from 183,293 to 504,755). When applied to the set of query molecules (the 745 approved drugs), we passed from the 8.3 single-protein targets that were on average known for an approved drug^7^ to the new estimation of 11.5 molecular targets when adding interactions with homologous proteins and protein complexes as well as more bioactivity data as explained above. An early estimate of the degree of drug polypharmacology is an average of 6.3 targets per drug based on data containing 5,215 drug-target associations and 557 targets^45^. Owing to using a more complete data set comprising 8,535 drug-target associations and 1,427 targets, we have now a more accurate estimation of the average number of targets hitting a drug, which almost doubles the best possible estimate nine years ago. Taken together, the results indicate that the true degree of drug polypharmacology is even higher than 11.5 targets per drug, as using more data should reveal in the future.

Lastly, while this study exploits the ChEMBL database to build and validate ligand-centric target prediction methods as well as their coupled reliability prediction method, any other database with target-annotated molecules of known chemical structure can also be used with this purpose (e.g. PubChem BioAssay^46^, SureChEMBL^47^ or BindingDB)^48^.

### How target prediction performance varies with a new knowledge-base

We have previously evaluated the performance of a simple target prediction method, MACCS fingerprints using dice score with k = 10 and a smaller knowledge-base, on a test set with 745 approved drugs^7^. Table 1 shows the corresponding results using the expanded knowledge-base allowing us to improve predictive performance. It is not trivial to anticipate how more data will affect the measured performance of the method because, among other unavoidable biases, drugs are more intensively studied than non-drug molecules^7^. However, the true performance of the method should improve as the target annotation of the database molecules becomes more comprehensive. The results of expanding the knowledge-base are shown in Table 1.

**Table 1:** Change in test set performance of the same ligand-centric target prediction method depending on the employed knowledge-base.

Method	avNPT	avACC	avPR	avRC	avMCC	avTN	avFP	avFN	avTP
Peon *et al*.^7^	7.9	0.996	0.296	0.403	0.300	3016.8	5.9	6.3	2.0
This paper	11.4	0.996	0.311	0.384	0.305	4186.1	8.4	8.5	3.0

The expanded knowledge-base can be found in the last row.

We can see that using the expanded knowledge-base translates to a substantial increase in the number of predicted targets (11.4 vs 7.9), which increases the usefulness of the method. In terms of predictive performance, we can observe that both correct and incorrect predictions increase (last four columns in Table 1), although the overall performance given by avMCC is slightly better when the method uses more data (0.305 vs 0.300). We also repeated the calculation using the Tanimoto score instead of the Dice score and appreciate no difference between both sets of results. Thus, we decided to employ the more commonly used Tanimoto score for the rest of the study.

### Benchmarking chemical similarity methods for ligand-centric target prediction

In this subsection, we investigate which is the optimal target prediction method by testing a suite of chemical structure fingerprints in combination with the Tanimoto score. From now on, we exclusively use the expanded knowledge-base, as this provides the largest number of predicted targets (i.e. avNPT values).

We started by looking for the optimal value for the k parameter (the number of top hits whose database annotation is used to predict targets). We considered k = 5,10,20,25 because at k = 25 the avMCC of the method has strongly declined (data no shown). As avNPT increases with growing k, we selected the lowest k for which avNPT is greater than avNKT (the average number of known targets across query molecules). If k was smaller than 10, avNPT < avNKT and thus the method would generally predict fewer targets that are known for the target. If k was higher, the performance would tend to be worse as target inferences would be drawn from increasingly less similar database molecules. Using the top 10 hits to predict targets provides the best compromise between these conflictive objectives on average across query molecules.

The results of these ligand-centric methods, all using k = 10, are shown in Table 2 and are sorted by descending avMCC value. The avACC values are close to the maximum value for all methods. As this is due to correctly classifying the very large number of true negatives, avACC is not very useful in practice. Based on the other metrics, methods employing Morgan fingerprints (ECFP-like) lead to better results than those with FeatMorgan fingerprints (FCFP-like), RDKit fingerprints (Daylight-like) or MACCS fingerprints (SMARTS-based implementation of the 166 public MACCS keys).

**Table 2:** Performance of the tested ligand-centric methods averaged over query molecules sorted by descending avMCC value.

Method	avNPT	avACC	avPR	avRC	avMCC
Morgan_hashed_bv_2_2048	11.7	0.996	0.348	0.423	0.339
Morgan_hashed_bv_2_512	11.7	0.996	0.345	0.421	0.337
Morgan_hashed_bv_2_1024	11.6	0.996	0.345	0.42	0.336
Morgan_bv_2_2048	11.7	0.996	0.342	0.424	0.335
Morgan_hashed_bv_3_512	11.5	0.996	0.346	0.416	0.334
Morgan_hashed_bv_3_1024	11.7	0.996	0.344	0.417	0.334
FeatMorgan_bv_3_512	11.6	0.996	0.347	0.416	0.332
Morgan_bv_2_1024	11.6	0.996	0.341	0.42	0.332
FeatMorgan_bv_3_2048	11.6	0.996	0.345	0.416	0.332
Morgan_hashed_bv_3_2048	11.7	0.996	0.342	0.414	0.332
FeatMorgan_bv_2_2048	11.7	0.996	0.345	0.418	0.331
FeatMorgan_bv_3_1024	11.6	0.996	0.346	0.415	0.331
Morgan_bv_2_512	11.7	0.996	0.341	0.415	0.331
Morgan_bv_3_2048	11.5	0.996	0.343	0.413	0.331
Morgan_bv_3_512	11.5	0.996	0.34	0.412	0.329
FeatMorgan_bv_2_1024	11.7	0.996	0.343	0.414	0.329
Morgan_bv_3_1024	11.5	0.996	0.34	0.41	0.328
FeatMorgan_bv_2_512	11.8	0.996	0.342	0.415	0.328
RDKit_2_7_2048_2	11.8	0.996	0.34	0.4	0.323
RDKit_2_7_1024_1	11.7	0.996	0.336	0.396	0.319
RDKit_2_7_2048_3	11.9	0.996	0.333	0.395	0.318
RDKit_2_7_1024_2	11.5	0.996	0.331	0.392	0.316
MACCS keys	11.4	0.996	0.311	0.384	0.305

Each method is named after the employed fingerprint, as the remaining components are common to all methods.

The method employing the Morgan hashed bit vector fingerprint with radius 2 and 2048 bits (*Morgan_hashed_bv_2_2048*) obtains the best results (avMCC = 0.339). However, the difference between the best- and worst-performing method is rather small (avMCC 0.339 vs 0.305). The avRC is relatively low (0.423), although this is partly due to data completeness (e.g. top hits were not tested for the same targets as the query molecule). It is also important to note that a ligand-centric methods can exhibit a large variability in RC depending on the query molecule^7^. The avPR represents a large hit rate of 34.8%. More so if we take into account that a false positive occurs when the predicted target is not known to be a target of the query molecule, which is not at all the same as not being a target of that molecule (99.96% of all the possible ligand-target pairs in the knowledge-base do not have measured bioactivity values). Overall, these results are remarkable given that approved drugs are much harder to predict than non-drug query molecules^7^ and the unusually large coverage provided by this type of methods (4,167 targets).

We also considered the alternative approach of using a similarity cutoff to determine the most similar database molecules with which to form the set of predicted targets for the query molecule (instead of using the top 10 most similar molecules). Table 3 shows the results for the best method in Table 2 (*Morgan_hashed_bv_2_2048*) using similarity cutoffs 90%, 80%, 70%, 60% and 50%. nNullQueries is the number of query molecules for which no hits are found (i.e. no database molecule obtains a similarity score higher than the employed cutoff and thus targets cannot be predicted for these drugs). In contrast, nQueries is the number of query molecules for which at least a hit is found (thus, performance is now averaged over nQueries). From these results, we can see that a 60% cutoff provides the best performance (avMCC = 0.338 leaving 69 drugs without predicted targets), which is slightly worse than that from using the top 10 most similar hits (avMCC = 0.339 leaving no drugs without predicted targets). Taking all these experiments into account, we use for the rest of the study the *Morgan_hashed_bv_2_2048* fingerprint along with the Tanimoto score based on the top 10 most similar hits to predict targets of a given query molecule.

**Table 3:** Performance of the best method in Table 2 (Tanimoto score on Morgan_hashed_bv_2_2048 fingerprints) using now similarity cutoffs 90%, 80%, 70%, 60% and 50% instead of the top 10 hits.

*Cutoff* (%)	*nNullQueries*	*nQueries*	*avNHITS*	*avNPT*	*avMCC*
90	347	398	2.22	5.07	0.243
80	258	487	4.13	5.69	0.295
70	151	594	9.51	8.06	0.333
60	69	676	21.03	11.66	0.338
50	30	715	54.82	21.43	0.323

nNullQueries is the number of query molecules for which no hits are found. In contrast, nQueries is the number of query molecules for which at least a hit is found (thus performance is now averaged over nQueries). AvNHITS is the average number of database molecules with similarity scores above the cutoff. The 60% cutoff provides the best performance (avMCC = 0.338 leaving 69 drugs without predicted targets), which is slightly worse than that from using the top 10 most similar this (avMCC = 0.339 leaving no drugs without predicted targets).

### Predicting the reliability of drug-target interaction predictions

We have thus far assessed the performance of a panel of ligand-centric target prediction methods spanned by considering four molecular similarity techniques and their variants. In this section, we introduce and validate a score to estimate the reliability of a predicted target. Every predicted target is by construction a known target of l of the 10 most similar molecules to the query molecule, where l ranges from 1 to 10. Our hypothesis is that the likelihood of a predicted target being a true positive will be higher if a higher proportion of top hits bind that target. Thus, we define the reliability score L of a ligand-target interaction prediction as L = l/10 (L = 0.1 predictions will be assigned minimum reliability, whereas L = 1 predictions will be regarded as the most reliable).

Table 4 presents the results of investigating whether this hypothesis holds using the best method identified in the previous section (see Table 2). Once the queries were carried out, the resulting set of ligand-target interaction predictions was partitioned into 10 categories according to their reliability scores. As expected, the proportion of true positives is strongly correlated with the predicted reliability L, which demonstrates the practical importance of this score.

**Table 4:** True-positive and false-positive target predictions for the test set of 745 approved drugs grouped by the reliability score L.

*L*	*TP*	*FP*	*TP*/*FP*	%*TP*	%*FP*
0.1	1,080	4,634	0.2	19%	81%
0.2	399	1,000	0.4	29%	71%
0.3	267	378	0.7	41%	59%
0.4	163	154	1.1	51%	49%
0.5	123	65	1.9	65%	35%
0.6	77	39	2.0	66%	34%
0.7	58	25	2.3	70%	30%
0.8	74	12	6.2	86%	14%
0.9	53	5	10.6	91%	9%
1.0	74	7	10.6	91%	9%

From L ≥ 0.4, TP is higher than FP. Importantly, %TP is strongly correlated with the reliability score L.

**Figure 2:**
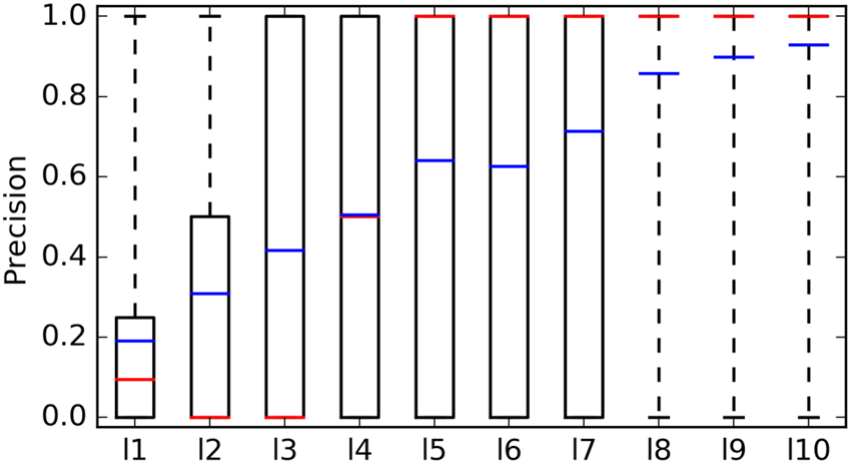
Boxplot showing how the precision of predicted targets varies depending on L (NB: l1 is l = 1 and corresponds to L = 0.1). The mean precision for a given L is marked with a blue line, whereas the median is given by a red line.

Next, we investigate which is the precision associated to a prediction depending on its reliability score. Figure 2 demonstrates that the average precision of predictions with a given L increases with the value of L. A large variability across query molecules is observed in the first seven groups of target predictions. From L = 0.8, this variability is strongly reduced, as it is increasingly harder to find false positives at those levels of reliability.

To the best of our knowledge, this is the first validated reliability score for ligand-centric target prediction methods and, as such, it is of great practical importance. Without acquiring further data, we can increase the hit rate of experimental validations by only testing the most reliable predictions. For instance, Fig. 2 shows that a target predicted with L = 0.4, i.e. 4 out of the 10 most similar molecules are annotated with this target, will be on average a true target of the query molecule 50% of the times. By contrast, L = 0.1 are only expected to obtain a hit rate of about 20%. Therefore, by prioritising L = 0.4 predictions over L = 0.1 predictions we should obtain 2.5 times more true positives in confirmatory assays.

Table 5 quantifies the main results visualised in Fig. 2. For instance, there are 81 target predictions of type L = 1 in 70 of the 745 queries. Out of these 70 queries, there are only 5 approved drugs for which targets were predicted with zero precision. For the remaining 65 query drugs, L = 1 target predictions achieved perfect precision (PR = 1). Therefore, the average precision over the 70 queries containing L = 1 is 0.929. This means that the targets predicted for an approved drug with maximum reliability should be true targets in 92.9% of the cases. This represents a large improvement over the 34.8% hit rate that would be expected if a predicted target is selected for testing without using the reliability score (Table 2).

**Table 5:** Performance results for the best method (quantification of results from Fig. 2).

*l*	*L*	*N*° *query molecules*	*N*° *Drug- target predictions*	*MeanPR*	*MedianPR*
1	0.1	662	5,714	0.191	0.1
2	0.2	514	1,399	0.309	0.0
3	0.3	362	645	0.417	0.0
4	0.4	228	317	0.505	0.5
5	0.5	142	188	0.641	1
6	0.6	95	116	0.626	1
7	0.7	65	83	0.714	1
8	0.8	77	86	0.857	1
9	0.9	49	58	0.898	1
10	1.0	70	81	0.929	1

The mean and median values for precision (PR) are shown, as well as the number of query molecules with a given l value.

The results in Table 5 clearly show that the reliability score L, calculated without using the true targets known for the query molecule, is highly predictive of how well these true targets are predicted. In other words, the higher L is, the more likely the predicted target is to be a true target (i.e. higher MeanPR).

We cannot stress enough that all the predicted targets of a molecule are provided by the method and therefore any of the considered 4,167 targets can be returned. This is because chemical structure similarity of target-annotated molecules to the query molecule is the only factor that controls which targets are predicted for a given query molecule. In particular, the number of known ligands for a target does not have any influence on which targets are predicted.

### Case studies

To better illustrate the strengths and limitations of this new method, we focus on the target predictions that are predicted to be most reliable (L = 1). From Table 5, we see that there are 81 targets predictions predicted with maximum reliability, of which 7 are flagged as possible false positives and 74 were confirmed as true positives.

These seven false-positive target predictions were made for five query drugs. The first of these drugs is Solifenacin succinate (CHEMBL1200803), which is the succinate salt of its first molecular hit (CHEMBL606901) and therefore both compounds should have the same targets. Three targets are annotated in all its hits, which are *Rattus norvegicus* Muscarinic acetylcholine receptors M1 (CHEMBL276), M2 (CHEMBL309) and M3 (CHEMBL320). On the other hand, the targets of this drug are *Homo sapiens* Muscarinic acetylcholine receptors M1 (CHEMBL216), M2 (CHEMBL211) and M3 (CHEMBL245). Thus, it is very likely that the hits will also bind the highly similar human homologues of these receptors (sequence identity ranging from 91.7% to 98.7%). In that case, these three predicted targets would become true targets of Solifenacin succinate.

Second, Neostigmine (CHEMBL278020) and its bromine salt (CHEMBL54126) are the query molecule and its first hit, respectively (again both compounds contain the same molecule and hence should have the same targets). Its top hits, but not Neostigmine, are all annotated to bind *Homo sapiens* acetylcholinesterase (CHEMBL220). Hence this is apparently a false-positive target prediction. However, *Torpedo californica* acetylcholinesterase (CHEMBL4780) is annotated as a target of Neostigmine and the mechanism of action of this clinically approved drug according to the CHEMBL database is “Acetylcholinesterase inhibitor”. Therefore, Neostigmine should also bind to the predicted target.

The next query drugs are Citalopram (CHEMBL1200781) and Escitalopram (CHEMBL1200322), which are the racemic form and its *S* enantiomer respectively. All the top hits of each of these drugs bind *Rattus norvegicus* Serotonin transporter (CHEMBL313). Visual inspection of both query molecules and their top hits revealed the very high degree of similarity between these, which indicates that the predicted target is very likely to be a true target of these two drugs. The latter is further supported by the binding of both drugs to human Serotonin transporter (CHEMBL228), with the *S* enantiomer being more potent than the racemic mixture.

Taking all this into account, it is reasonable to think that these six apparent false positives will be revealed as true targets of their respective drugs once tested.

**Figure 3:**
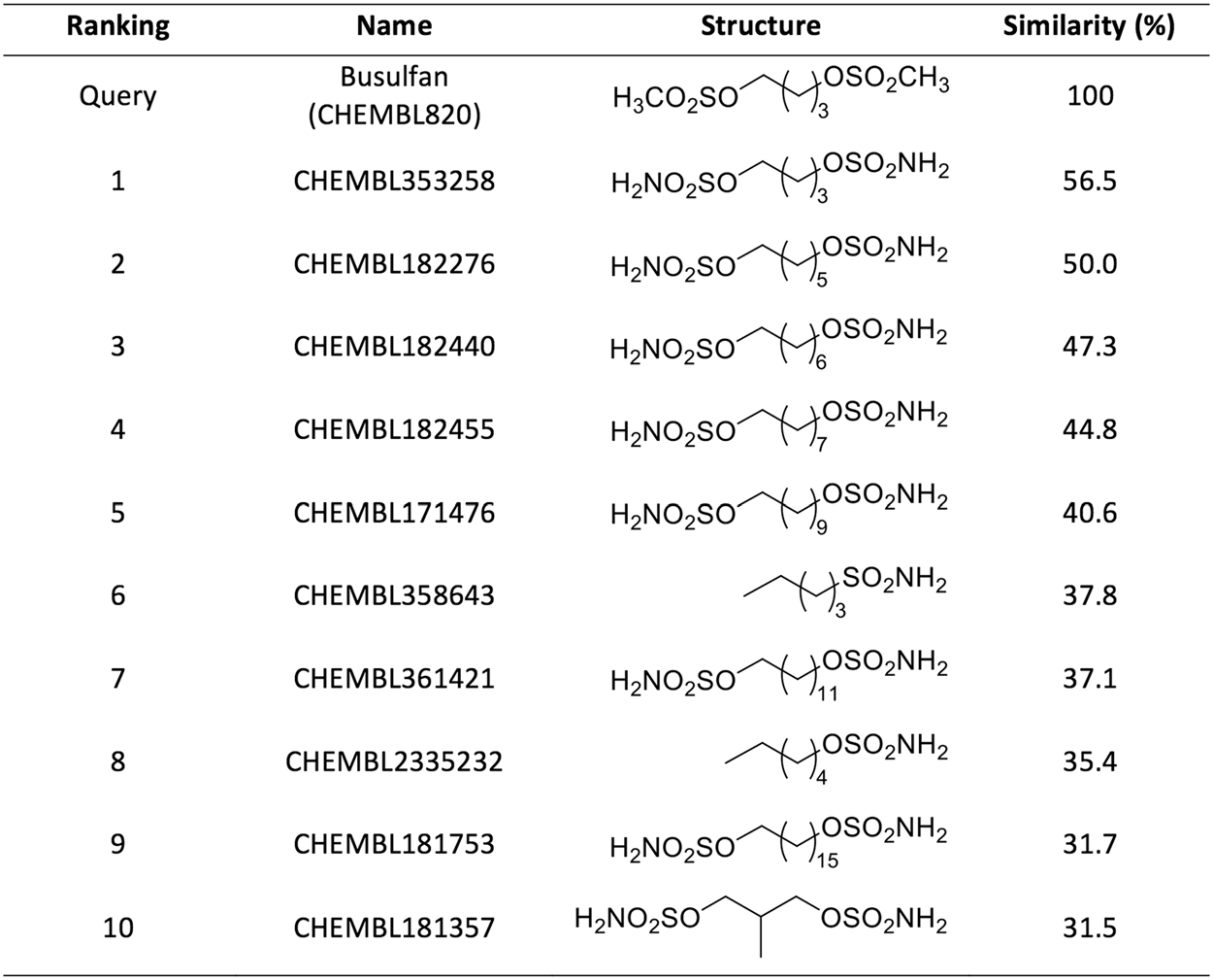
The top 10 hits for the Busulfan query are shown ranked by similarity. The approved drug Busulfan (ChEMBL820) is an alkylating agent. All these hits bind to the predicted target, Carbonic anhydrase 2 (target CHEMBL205), and hence this is a L = 1 target prediction. Since Carbonic anhydrase 2 is not a known target of Busulfan, this is one of the seven L = 1 false positives. This seems to be a genuine false positive due to the relatively low similarity of the hits to the query molecule (56.5–31.5%).

Next, we study the last of these L = 1 false positives in more detail. This is the prediction of *Homo sapiens* Carbonic anhydrase II (CHEMBL205) as a target of Busulfan (CHEMBL820). While the top 10 hits of Busulfan bind this enzyme (Fig. 3), Busulfan itself is not annotated as one of the ligands of this target. Busulfan is an alkylating agent used in cancer therapy that forms DNA-DNA intra-strand cross-links between the DNA bases guanine and adenine and between guanine and guanine through a SN2 reaction with mesylate groups -OSO_2_CH_3_ as leaving groups^49^. Mesylate groups are not present in any of the top hits, which are much more similar among them than with Busulfan. Therefore, this seems to be a genuine false positive.

**Figure 4:**
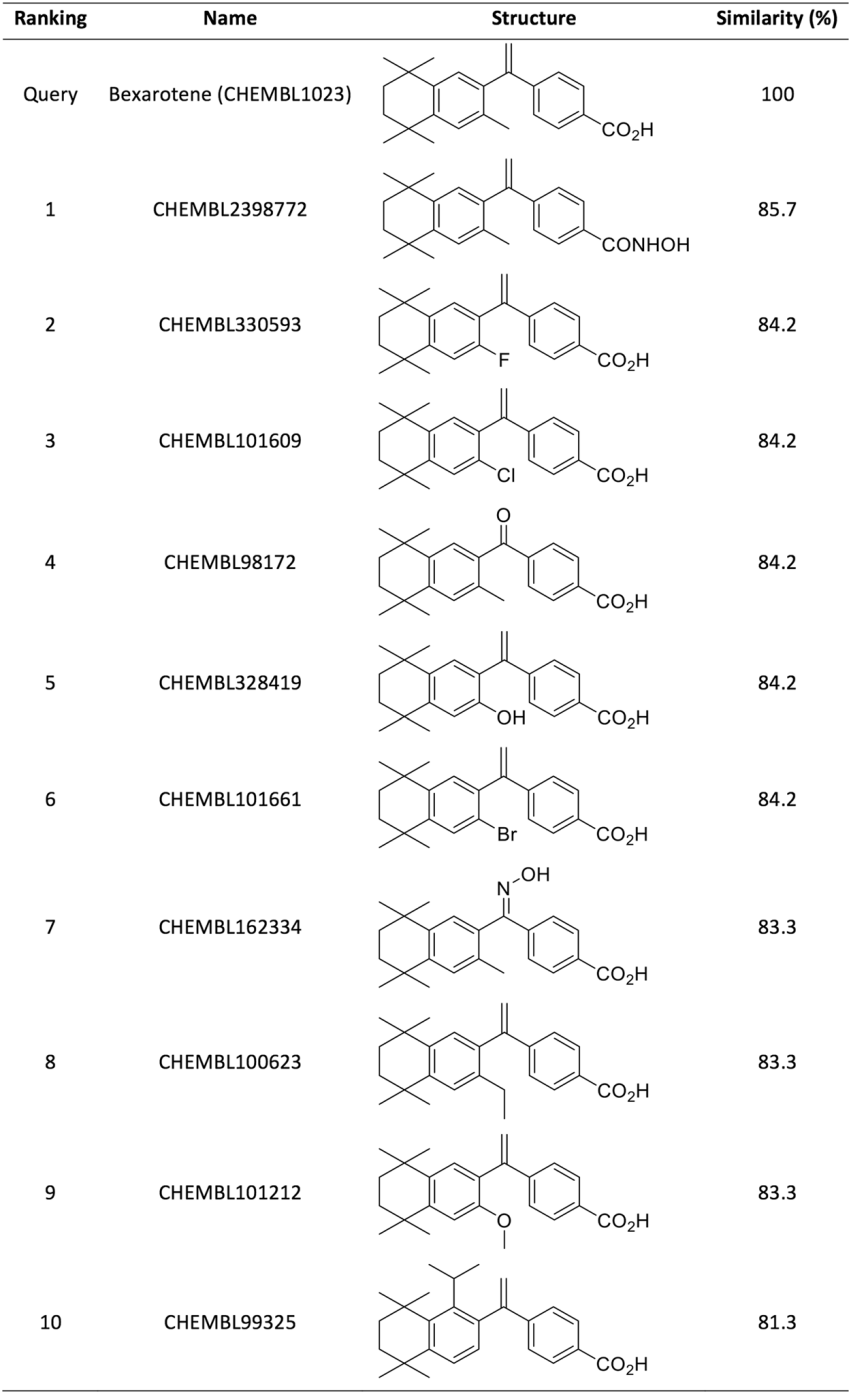
Chemical structures of the 10 most similar database molecules to Bexarotene. This is a L = 1 true positive prediction as these molecules bind the predicted target (Retinoid X receptor alpha).

Having analysed L = 1 false-positive target predictions, we turn our attention to confirmed L = 1 true positives. Figure 4 shows a representative example of a L = 1 true-positive target prediction. This is for Bexarotene (CHEMBL1023), an antineoplastic agent indicated by the FDA for Cutaneous T cell lymphoma. We can see that the query molecule and its top 10 hits are close derivatives of the same core scaffold and hence are highly similar among them. Each of these hits binds the human Retinoid X receptor alpha (CHEMBL2061) and thus this target is predicted for Bexarotene with maximum reliability. Since Bexarotene is a Retinoid X receptor agonist, this is a true-positive target prediction.

## Conclusion

We have introduced and rigorously tested a suite of ligand-centric methods for target prediction exploiting an unusually large knowledge-base (887,435 ligand-target pairs, 504,755 database molecules and 4167 macromolecular targets). From here, we have verified that an approved drug has on average 11.5 known targets below 10 µM. This represents a substantially higher degree of drug polypharmacology than what is obtained using substantially less data^7, 45^.

We also found that the target prediction methods using the 10 most similar molecules to the query based on the Morgan hashed bit vector fingerprint radius 2 and 2048 bits provides the best results. However, the difference between the best- and worst-performing method is rather small. While the average recall across queries of this method is relatively low (0.423), its achieved average precision represents a large hit rate of 34.8% with 11.7 predicted targets per query. This is a remarkable performance taking into account that approved drugs are much harder to predict than non-drug query molecules^7^ and the unusually large coverage provided by this type of methods (4,167 targets). Having said this, it is possible that other types of molecular similarity lead to more accurate target prediction methods or at least to complementary methods predicting targets missed by those presented in this study. For example, the drug discovery process often generates a large number of molecules with a common active chemical scaffold in order to determine the structure-activity relationship. These derivatives of the active scaffold are not necessary similar in terms of global molecular similarity. Therefore, partial molecular similarity with a clinical drug as query may be able to identify targets annotated in such derivatives, but missed by ligand-centric target prediction based on global molecular similarity.

Most importantly, we have introduced and validated a first-in-kind method to predict the reliability of ligand-centric target predictions. Without exploiting further data, we can increase the hit rate of experimental validations by only testing the most reliable predictions. In particular, we have estimated that the targets predicted for an approved drug with maximum reliability should be true targets in 92.9% of the cases. This represents a large improvement over the 34.8% hit rate that would be expected if the reliability score is not used. This validation shows that the reliability score L can be employed as the probability of the target prediction being correct.

We have also investigated why 7 of the 81 targets predictions estimated to be predicted with maximum reliability (L = 1) are flagged as possible false positives. These 7 L = 1 false-positive target predictions are for five query drugs: Solifenacin, Citalopram, Escitalopram, Neostigmine and Busulfan. Solifenacin, Citalopram and Escitalopram are annotated with the human homologue of the predicted target, whereas the hits are annotated with the murine homologue of that target. Conversely, a homologue of the Neostigmine’s L = 1 predicted target is known to be a target of this drug. Owing to the high sequence identity between these sets of homologue proteins, it is highly likely that these six apparent false positives will become targets of their respective drugs once tested. By contrast, the seventh false positive seems genuine, as the query drug Busulfan has relatively low similarity to its top hits, much more similar among them. On the other hand, the remaining 74 drug-target associations predicted with L = 1 were confirmed as true positives. We discussed Bexarotene as a representative example of such L = 1 true-positive target predictions, which are characterised by the very high molecular similarity between these query molecules and their corresponding top 10 hits.

In practice, the performance of the method should be actually higher due to the ligand-target bioactivity matrix being sparsely populated (only 0.04% of all the possible ligand-target pairs in the knowledge-base have at least a bioactivity value associated). Indeed, a false positive occurs when the predicted target is not known to be a target of the query molecule, which is not at all the same as not being a target of that molecule. Data sparsity negatively affects the precision of the method in cases where the predicted target is a true target of the drug, but no bioactivity value is available for this drug-target pair. Data sparsity negatively affects the recall of the method when a known target of the query drug is not predicted because the bioactivity of its top hits for that target has not been determined yet. Consequently, the reported performance must be regarded as a worst-case scenario and thus we conclude that this method should work better in prospective validations than here estimated.

## Methods

### Data provenance

We downloaded release 20 of the ChEMBL database^43^ as a PostgreSQL dump, which contains data for 10,774 targets, 1,456,020 ligand molecules with disclosed chemical structure and 13,520,737 bioactivities curated from 59,610 scientific publications. Relevant data was retrieved using PostgreSQL 9.4.3 queries and all further processing was done with Python 2.7.9. As usual, single-atom fragments were removed and the largest molecular fragment of each compound in a salt form was kept. Next, we generated two separated tables from this data set, one for the query molecules and another for database molecules, each with their canonical SMILES, ChEMBL IDs and annotated targets.

### Definitions of target and known ligand-target association

As discussed in the subsection “Expanding ligand-target knowledge-base”, a more inclusive definition of known ligand-target association than previously used^7^ was employed here. Specifically, the following filters were applied to identify known ligand-target associations from the downloaded data: (i) the activity value for the ligand-target pair had to be better than 10 µM using relations “=” or “<” (for target-ligand pairs with multiple activity values, the lower value was used), (ii) the assay type was either binding or functional, (iii) the bioactivity type was IC_50_, K_i_, EC_50_, K_d_ or Potency (Potency comprises IC_50_, EC_50_, AC_50_, GI_50_ or K_i_ presented in PubChem^46^ repositories and marked up as an active concentration from a confirmatory assay) and (iv) the target confidence score was at least 7. The latter means that only molecular targets were considered, i.e. those with direct protein complex subunits assigned, homologous single protein target assigned and direct single protein target assigned (confidence score 7, 8 and 9, respectively).

### Generating data sets for the benchmark

These data sets are derived from the expanded knowledge-base. To study how method performance changes with the definitions of known targets, we focused on the same 745 approved drugs that were used as query molecules (test set) in a previous study^7^. These molecules collectively contain 1,427 targets spanning 8,535 drug-targets pairs.

The database molecules (training set) were formed by all the molecules passing the filters above except for the 745 drugs, which were removed to avoid any overlap between query and database molecules. After filtering, there were 504,755 database molecules with a total of 4,167 known targets coming from 887,435 known ligand-target associations.

### Tested target prediction methods

Each ligand-centric target prediction method differently measures the similarity of the query molecule to a very large set of target-annotated molecules (the database molecules). Such similarity is defined by the adopted description of the chemical structure of each molecule (the fingerprint). The fingerprints available at RDKit (http://www.rdkit.org/) were used as the basis of this similarity calculation: Morgan fingerprints and FeatMorgan fingerprints^50^ (ECFP- and FCFP-like fingerprints, respectively) with different radius (2 and 3) and encoding lengths (512, 1024 and 2048 bits); HashedMorgan fingerprint with 2048 bits and radius 2; RDKit fingerprints with minimum and maximum numbers of bonds (2 and 7, respectively) using 1024 and 2048 bits and number of bits per hash 1, 2 or 3; and MACCS keys.

Tanimoto score was used to measure the similarity between these fingerprints:

1 $$Tc=\frac{c}{(a+b+c)}$$

where a and b are the number of ON bits of the molecular fingerprints A and B, and c is the number of bits in common between both fingerprints. The Dice score was also used to compare in a prior method^7^:

2 $$Dice=\frac{2c}{(a+b)}$$

In terms of efficiency, predicting the targets of a query molecule exploiting this knowledge-base of 887,435 ligand-target associations takes about 30 seconds using a single CPU core. We could therefore comfortably calculate target predictions for our test set of 745 query molecules in this way and thus we were not required to implement parallel computation to enable the use of multiple CPU cores.

### Measuring classification performance

The performance of each method was evaluated with the following metrics: accuracy (ACC), precision (PR), recall (RC), Matthews Correlation Coefficient (MCC) the Number of Predicted Targets (NPT). These metrics were introduced in the subsection “Experimental design”. Here we state their expressions:

3 $$ACC=\frac{(TP+TN)}{(TP+FP+TN+FN)}$$

4 $$PR=\frac{TP}{(TP+FP)}$$

5 $$RC=\frac{TP}{(TP+FN)}$$

6 $$MCC=\frac{(TP\times TN-FP\times FN)}{\sqrt{((TP+FP)(TP+FN)(TN+FP)(TN+FN)}}$$

7 $$NPT=TP+FP$$

### Data availability statement

The data used for this study is freely available at https://www.ebi.ac.uk/chembl/.
